# Design X Bioinformatics: a community-driven initiative to connect bioinformatics and design

**DOI:** 10.1515/jib-2022-0037

**Published:** 2022-07-22

**Authors:** Björn Sommer, Daisuke Inoue, Marc Baaden

**Affiliations:** School of Design, Royal College of Art, London, UK; Faculty of Design, Kyushu University, Fukuoka, Japan; Université Paris Cité, CNRS, Laboratoire de Biochimie Théorique, 13 rue Pierre et Marie Curie, F-75005, Paris, France

**Keywords:** biodesign, design processes, microscopy, networks visualization, standardization

## Abstract

Bioinformatics applies computer science approaches to the analysis of biological data. It is widely known for its genomics-based analysis approaches that have supported, for example, the 1000 Genomes Project. In addition, bioinformatics relates to many other areas, such as analysis of microscopic images (e.g., organelle localization), molecular modelling (e.g., proteins, biological membranes), and visualization of biological networks (e.g., protein–protein interaction networks, metabolism). Design is a highly interdisciplinary field that incorporates aspects such as aesthetic, economic, functional, philosophical, and/or socio-political considerations into the creative process and is usually determined by context. While visualization plays a critical role in bioinformatics, as reflected in a number of conferences and workshops in the field, design in bioinformatics-related research contexts in particular is not as well studied. With this special issue in conjunction with an international workshop, we aim to bring together bioinformaticians from different fields with designers, design researchers, and medical and scientific illustrators to discuss future challenges in the context of bioinformatics and design.

## 1 Introduction

Bioinformatics is a field that applies computer science approaches to the analysis of biological data. It is widely known for its genome-based analysis approaches that have supported, for example, the 1000 Genomes Project. However, it also relates to many other areas, such as analysis of microscopic images (e.g., organelle localization), molecular modelling (e.g., proteins, biological membranes), and visualisation of biological networks (e.g., protein-protein interaction networks, metabolism).

Since design is a highly interdisciplinary field, aspects such as aesthetic, economic, functional, philosophical, and/or socio-political considerations enter into the creative process and are usually determined by context. While visualisation and visual approaches play a crucial role in bioinformatics, as reflected in a number of conferences and workshops in the field, this is not yet the case for design, especially in bioinformatics-related research contexts.

With this special issue, in conjunction with an international workshop initiative, we aim to bring together bioinformaticians from different fields with designers, design researchers, and medical and scientific illustrators to discuss future challenges in the context of bioinformatics and design: http://desxbioinf.i2d.uk/.

Because bioinformatics is a field that often combines abstract and spatial data and frequently considers standardization approaches, it offers a number of opportunities for future designers.

In addition, we would like to place Design X Bioinformatics in the context of the emerging field of Biodesign, which uses the biological capabilities of organisms to meet specific user needs, for example, in terms of materials development, synthetic biology applications, or the creation of bio-digital hybrid systems. In such systems, both the digital and biological components are engineered to work together.

### 1.1 Topics

For this initiative, and thus for this special issue, contributions were solicited from a variety of areas that combine bioinformatics and design approaches. A non-exhaustive list of such potential topics includes:

– Challenges in Design X Bioinformatics
– Contextualization of previous work to the design field
– Investigating different areas of design (visualization, processes, SDGs – Sustainable Design Goals, service design) in the context of Bioinformatics
– Bio-related 3D Modelling and Design
– Generation of medical and scientific illustrations
– Visualization of biological phenomena (light microscopy, electron microscopy) and their abstraction

As the summary of research contributions below shows, a variety of these topics are covered, providing a snapshot of current research directions in the field.

### 1.2 Contextualization with respect to Biodesign and bio visualisation

Biodesign is an emerging topic for which a clear definition has not yet been found. Several challenges and workshops on Biodesign have been held in recent years [1–3]. The openness of the field allows for a high degree of creativity. Biodesign refers to both computational and synthetic biology and integrates the principles of human-centred design [2].

However, previous workshops have not yet addressed the relationship between design and bioinformatics in depth, often focusing on more general design issues related to biological systems.

There are already a number of workshops and conferences that are active in related areas and have a strong focus on visualization but are not yet closely related to design. In a non-exhaustive list, we briefly describe three such initiatives related to this special issue:

– The “Visualizing Biological Data (VIZBI)” conference has attracted – besides computer scientists and Bioinformaticians – in numbers of artists and designers in recent years [4]. This conference was launched in 2010 and provides an (almost) annual overview of new visualization approaches in various biological fields.
– The “Workshop on Molecular Graphics and Visual Analysis of Molecular Data” has promoted visual analysis of molecular data at the EuroVis conference (almost) annually since 2018 and also attracts papers with a minor design components [5].
– The workshop “From Virtual Reality to Immersive Bioinformatics” was organised in 2018 as part of the Stereoscopic Displays and Applications conference [6]. It explored various projects that were or are on their way to using virtual reality technologies to advance bio-visualization projects toward data analysis and decision making.

### 1.3 Design X Bioinformatics logo design

**Figure 1:**
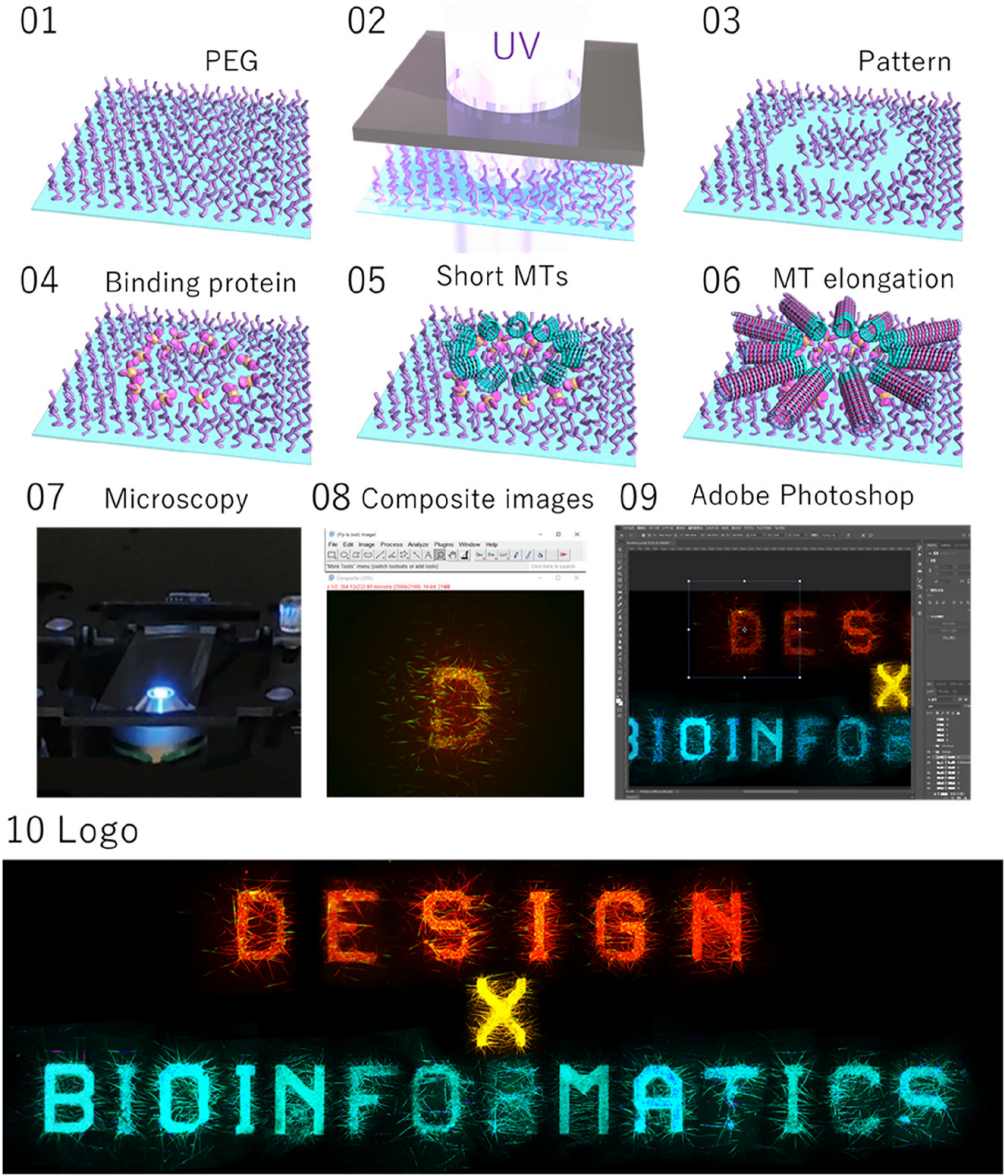
The creation of the workshop logo: the workshop logo was created using the micropatterning technique of microtubules (MTs) on a glass substrate grafted with polyethylene glycol (PEG) (steps 01 to 06). Partial removal of PEG by UV irradiation via a photomask enables patterning of microtubules on the glass surface. Images of the microtubule letter patterns were captured under a fluorescence microscope (step 07), followed by image processing in image J and adobe photoshop to create the final logo image (steps 08 and 09).

Figure 1 shows the logo design developed for this workshop. The process combined experimental design of a stylized header text with subsequent image post-processing to assemble the final design. The original experimental header text image was created using a micropatterning technique of microtubule cytoskeletons [7, 8].

Microtubules are critical for supporting cellular architecture, intracellular transport, and chromosome segregation. Microtubule self-assembly is an essential physical process underlying these cellular functions. The micro-patterning technique is an *in vitro* method to study and visualize the physical principle underlying the phenomenon of microtubule self-assembly by designing the structure of microtubule assemblies in a simplified and ideal form.

Designed *in vitro* systems such as microtubule micropatterning are already powerful tools for studying cellular phenomena observed under the microscope. However, microscopy has limitations in terms of the scale at which it can observe, and it is a challenging task to visualize cellular structures and phenomena at the molecular level. Recent computational approaches can visualize such phenomena at the molecular scale and break the limitations of wet lab experiments.

From another point of view, the various geometric patterns created by the micro-patterning technique using biomaterials (Figure 1) are a kind of bioartwork in which artificial and biological natures coexist, creating a sense of wonder not found in conventional art. Such bio art can attract the attention of people outside the research field and provide an opportunity for researchers from other fields to enter the field and collaborate.

Therefore, we chose the final image as the logo to promote the Design X Bioinformatics workshop, created by this *in vitro* approach.

## 2 Overview of the special issue

Of the nine manuscripts submitted for this special issue on design in the context of bioinformatics, five papers have been accepted for publication after peer review. We provide here an overview of the accepted submissions:

### 2.1 Integrative illustration of a JCVI-syn3A minimal cell

**Figure 2:**
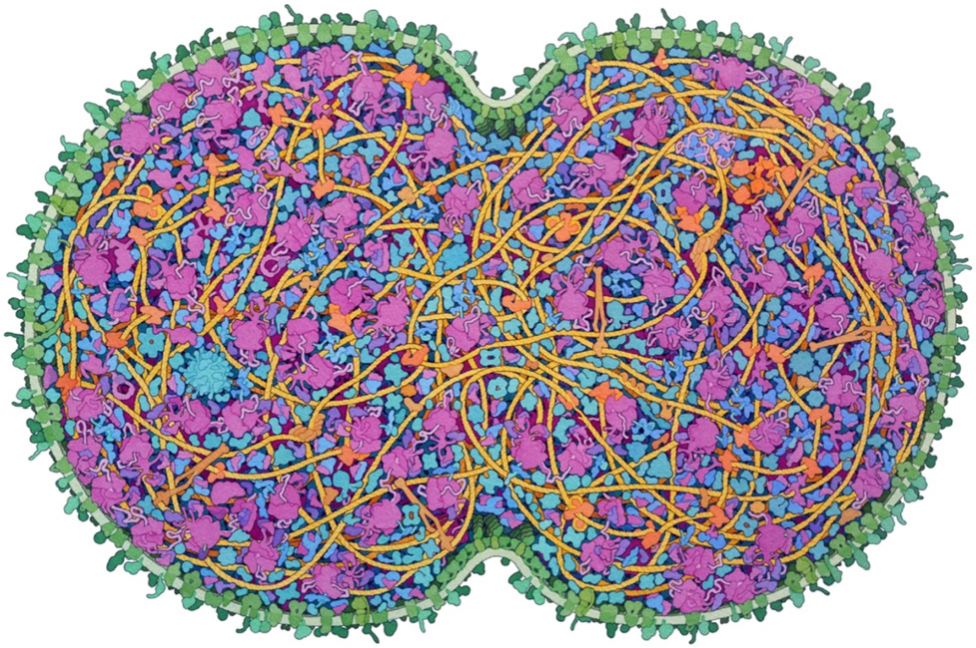
Integrative illustration of a JCVI-syn3A minimal cell: the artistic conception of a cross-section through a dividing cell. Small molecules, ions, and water have been omitted for clarity (©2022 Goodsell, CC BY 4.0 [9]).

In his keynote contribution, David Goodsell presents an approach to building a cross-sectional model of an entire JCVI-syn3.0 minimal cell (Figure 2). To this end, data from microscopy were combined with data from genomics, proteomics, and structural biology. Scale and color selection were optimized, and the complexity of the cell was appropriately reduced to generate enthusiasm for the underlying science while maintaining scientific accuracy. The final illustration integrates a number of functional narratives, including the division of the cell and the first representation of an entire cellular proteome. The article includes detailed discussions of design decisions, narrative and structural choices, modelling, renderings, and applications. In this way, David Goodsell offers both novices and visualization experts exclusive insights into how one of the most prolific life scientists and artists makes his design decisions [9].

### 2.2 Design considerations for representing systems biology information with SBGN

**Figure 3:**
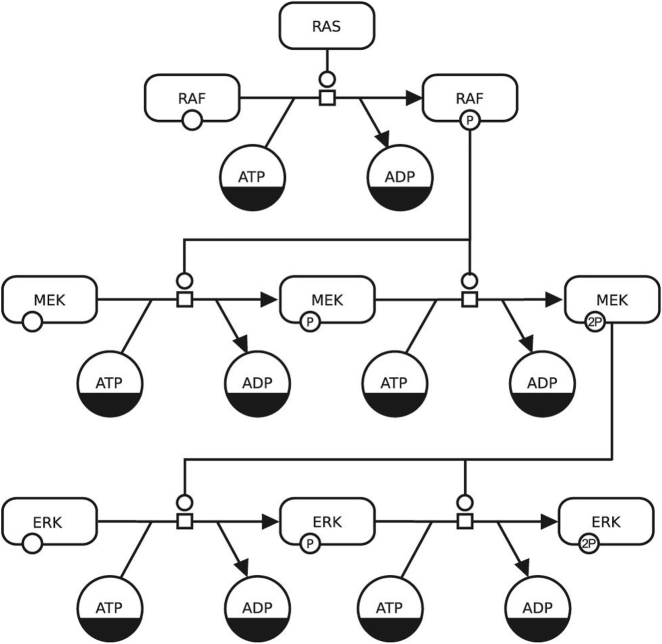
Design considerations for representing systems biology information with SBGN: an example of a SBGN PD (process description) map using two types of entity pool nodes; one for pools of different macromolecules and another for pools of simple chemicals (©2019–2022 Schreiber et al., CC BY 4.0 [10]).

This manuscript provides an overview of the design principles to consider when creating visualizations of systems biology information, using the Systems Biology Graphical Notation (SBGN) as an example. A review of the literature on the design of graphical representations in biology using the SBGN recapitulates previous work. The paper provides a detailed overview of the theoretical background, critically discusses the use and design of glyphs, criteria for good network layouts, and building blocks for SBGN PD maps (Figure 3). The principles, requirements, and design criteria presented are useful for creating such visualizations. Finally, the authors present a tool that supports the creation of systems biology visualizations and implements these design principles [10].

### 2.3 Design – a new way to look at old molecules

**Figure 4:**
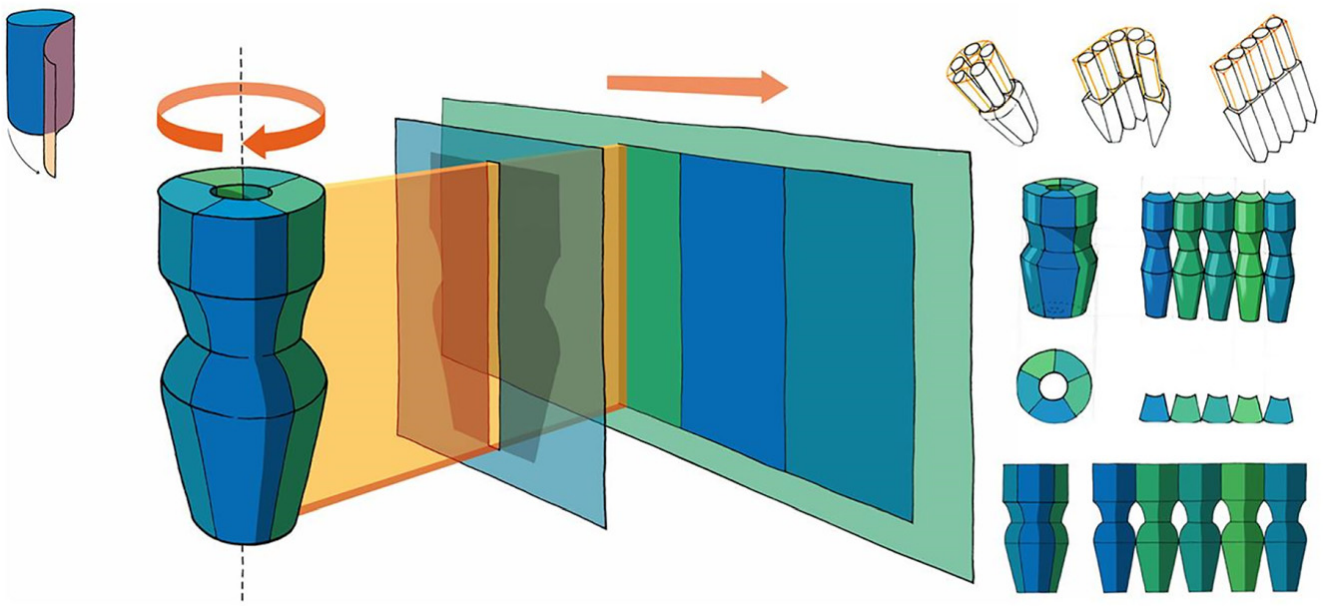
Design – a new way to look at old molecules: unrolling the surface – transfer of the photographic principle of 360-degree rotation of the camera to the case of taking a panorama of a protein surface. The final concept is drawn in the centre of the image, the first studies are shown smaller on the right side (©2022 Spalvieri et al., CC BY 4.0 [11]).

In this manuscript, the authors outline the importance of using design in structural biology and bioinformatics. First, the authors present practical examples of the benefits of researchers working with designers/illustrators and the collaborative process between the two. The fusion of art and science also new designs for visualising, representing, and understanding the invisible molecular world of proteins. Such designs go beyond molecular visualization and redefine the way we see the molecular world. Inspired by a panoramic camera, the authors propose a method to flatten the three-dimensional structure of proteins and convert it into a two-dimensional projection, which is then used to design information visualization for ion channels (Figure 4). This projection allows 2D imaging of the dynamic properties of the entire surface of ion channels without hidden segments and facilitates analysis of temporal evolution and comparison of surface properties. The authors point out that while accurate representation of the molecular structure of visualized proteins is a key element in scientific publications, comic-style graphic design is better suited for scientific communication aimed at a broader audience. Finally, the authors present several digital tools and approaches that can be used in scientific workflows to facilitate self-implementation by the designer or artist [11].

### 2.4 Colors in the representation of biological structures

**Figure 5:**
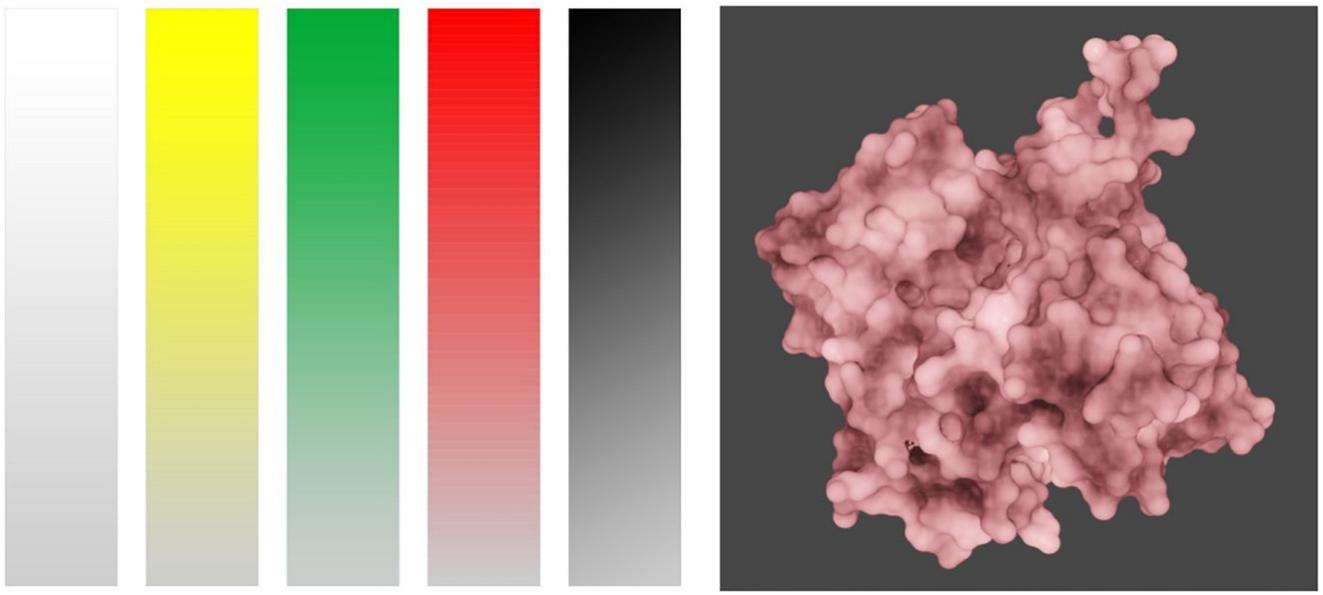
Colors in the representation of biological structures: molecular structures and their intensity – each color can have a range of intensity (left), which can be used to represent properties expressed by a continuum (right), here: an actin monomer (©2022 Zoppè et al., CC BY 4.0 [12]).

At the molecular level, structures such as proteins, sugars, nucleic acids, etc., have no color of their own. But to illustrate and explain the diversity and functionality of these cellular components, colors are often used. In this article, the author proposes a new initiative to help bring together biologists, artists, computer graphic designers, and perception experts to reconcile these two situations. The concept can be developed in a series of iterations that engage the community, including discussion panels, technical challenges, experimental studies, and outreach activities. Examples discuss, e.g., how color ranges can be used to encode intensities of molecular structures (Figure 5). A unique aspect of this paper is that it also discusses the cultural implications of color and its relation to molecular structures as well as crowded cells [12].

### 2.5 Considering best practices in color palettes for molecular visualisations

**Figure 6:**
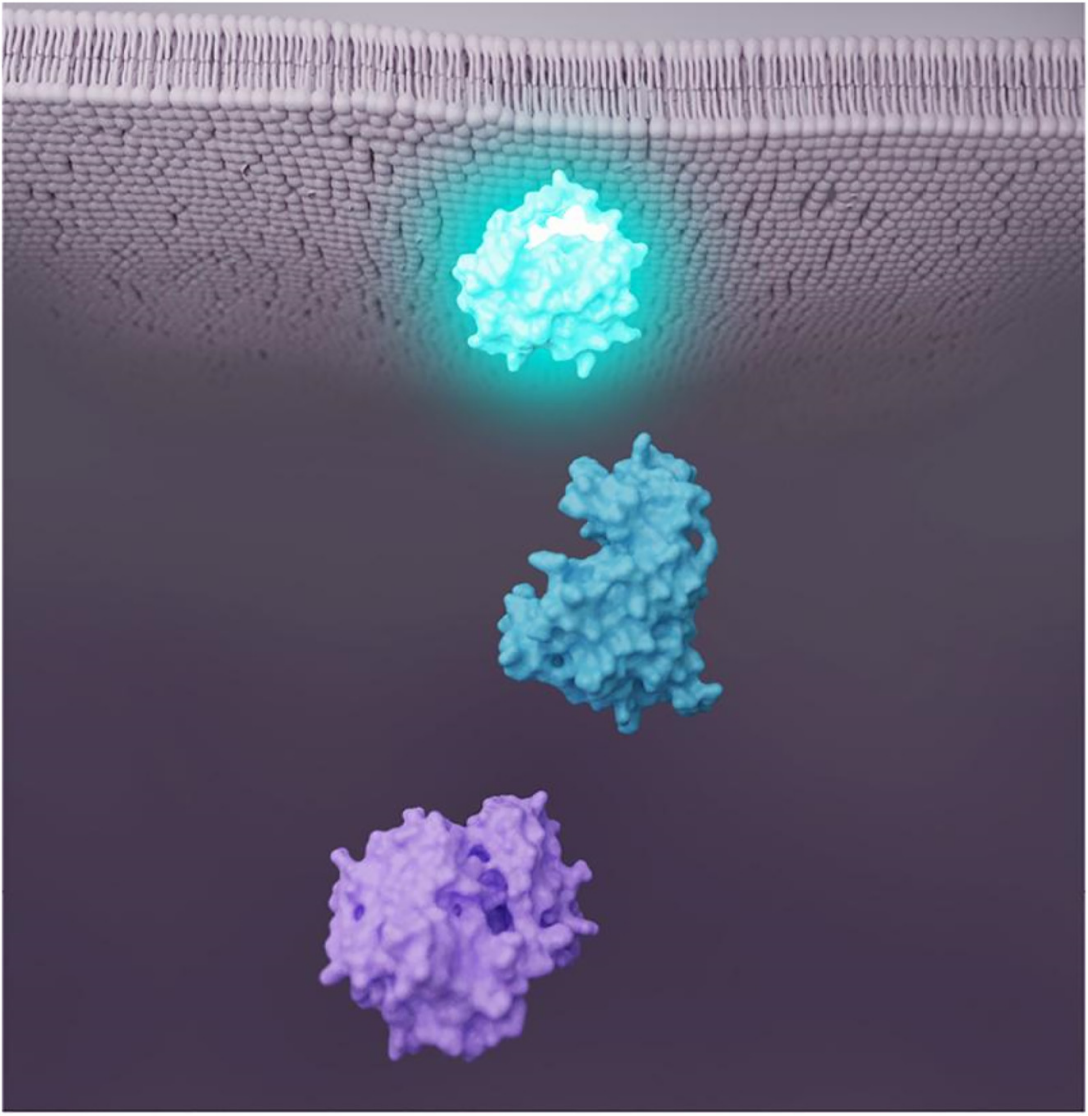
Considering best practices in color palettes for molecular visualisations: simple biomedical illustration depicting key molecules in an intracellular metabolic pathway – the color here helps to give functional meaning to the visualisation: similar colors show that the three molecules are linked, and a color progression indicates the order of the molecules in the pathway (©2022 Garrison et al., CC BY 4.0 [13]).

Garrison and Bruckner discuss the use of color in molecular visualizations, focusing on the reasons and problems associated with the broad and often arbitrary use of color. They provide background information and considerations that underscore the need for a color system for molecular visualizations. The guidelines offer the added benefit of simplifying the creation process for those unfamiliar with color theory. Best practise considerations relate to three key concepts – aesthetics, interpretability, and effectiveness. The challenges and perspectives described highlight the importance of involving multiple stakeholders and diverse perspectives in the development of such guidelines. The overall goal is to link colors better and more consistently to the functional or structural semantics of molecules (Figure 6). These efforts can lead to more interpretable and effective molecule visualisations without compromising aesthetics or creative freedom. An interesting discussion concerns the link between color techniques and interpretation [13].

## 3 Outlook

This special issue offers several examples of how the worlds of design and bioinformatics can be brought together to open new avenues of research and promote cross-fertilization between these disciplines. We can already see that there are some recurring themes, such as that of color, and that it is desirable to develop generally applicable design principles for the particular area where design and bioinformatics meet. There may currently be a lack of places where these communities naturally intersect, and the organization of workshops specifically focused on this area may be necessary to further advance such studies.

We have also begun to explore the relationship between Biodesign and Bioinformatics here. As mentioned earlier, the field of Biodesign is not yet clearly defined, but obviously Design X Bioinformatics Design shares a large cross-section with Biodesign. The flourishing of Biodesign offers great potential for the future development of the Design X Bioinformatics Design field.
